# Reference Intervals of Thyroid Function Tests Assessed by Immunoassay and Mass Spectrometry in Healthy Pregnant Women Living in Catalonia

**DOI:** 10.3390/jcm10112444

**Published:** 2021-05-31

**Authors:** José María Hernández, Berta Soldevila, Inés Velasco, Fernando Moreno-Flores, Laura Ferrer, Alejandra Pérez-Montes de Oca, Cecilia Santillán, Carla Muñoz, Sílvia Ballesta, Cristina Canal, Manel Puig-Domingo, María Luisa Granada

**Affiliations:** 1 Institut d’Investigació en Ciències de la Salut Germans Trias i Pujol, s/n Camí de les Escoles, 08916 Badalona, Spain; jmhernandez@igtp.cat; 2 Endocrinology & Nutrition Department, Hospital Universitari Germans Trias i Pujol, s/n Carretera del Canyet, 08916 Badalona, Spain; bsoldevila.germanstrias@gencat.cat (B.S.); laura.estopinan@gmail.com (L.F.); alec148@gmail.com (A.P.-M.d.O.); silvia.ballesta@gmail.com (S.B.); 3 Endocrine, Thyroid & Obesity Research Group, Institut d’Investigació en Ciències de la Salut Germans Trias i Pujol, s/n Camí de les Escoles, 08916 Badalona, Spain; inesvelas@msn.com (I.V.); carlaa.mj@gmail.com (C.M.); granadaybern@gmail.com (M.L.G.); 4 Gynecology & Obstetrics Department, Hospital Universitari Germans Trias i Pujol, s/n Carretera del Canyet, 08916 Badalona, Spain; 5 Pediatrics, Gynecology & Obstetrics Department, Autonomous University of Barcelona, Campus UAB, Plaça Cívica, 08193 Bellaterra, Spain; 6 Clinical Biochemistry Department, Hospital Universitari Germans Trias i Pujol, s/n Carretera del Canyet, 08916 Badalona, Spain; fernanmf81@gmail.com; 7 Endocrinology Department, Doctor Arturo Oñativia Hospital, 30 E.Paz Chain, Salta A4400AWQ, Argentina; ceciliasantillan3.0@gmail.com; 8 Gynecology & Obstetrics Department, ASSIR La Riera, Hospital Universitari Germans Trias i Pujol, 1 Riera Matamoros, 08911 Badalona, Spain; ccanal.mn.ics@gencat.cat

**Keywords:** thyroid function, pregnancy, reference intervals, immunoassays, tandem mass

## Abstract

Background: Recent guidelines recommend establishing a local reference interval (RI) for thyroid function. We aimed to establish trimester-specific RIs for thyrotropin (TSH) and free thyroxine (FT4) in a cohort of healthy pregnant women in Catalonia (Spain). Methods: A prospective observational study was conducted with 332 healthy pregnant women, from the first trimester (1T) to delivery. TSH was measured using an Architect^®^ immunoassay (Abbott) and FT4 by two immunoassays, Architect^®^ (Abbott) and Cobas^®^ (Roche), in the three trimesters. FT4 was also measured by liquid chromatography mass spectrometry (LC/MS/MS) in the 1T. Results: TSH (µUI/mL) increased throughout pregnancy (1T: 0.03–3.78; 2T: 0.51–3.53; 3T: 0.50–4.32; *p* < 0.0001) and FT4 (pmol/L) progressively decreased (Architect^®^ 1T: 10.42–15.96; 2T: 8.37–12.74; 3T: 8.24–12.49; *p* < 0.0001; and Cobas^®^: 1T: 11.46–19.05; 2T: 9.65–14.67; 3T: 8.88–14.54; *p* < 0.0067). The FT4 RI during 1T determined LC/MS/MS was 8.75–18.27. Despite the 1T FT4 results measured by LC/MS/MS and with the two immunoassays being significantly correlated, the results obtained by the three methods were found to be non-interchangeable. Conclusions: We established trimester-specific RIs for TSH and for FT4 with immunoassays in our population. We also validated the 1T FT4 using LC/MS/MS to confirm the results of FT4 lower than the 2.5th percentile or higher than the 97.5th percentile.

## 1. Introduction

Thyroid function is crucial for fetal growth and neurodevelopment throughout intrauterine life [1]. Thyroid dysfunction, especially hypothyroidism, can adversely affect pregnancy outcomes and fetal development [2]. However, the normal limits for thyroid function parameters during the different stages of pregnancy remain unclear. Various factors may influence thyroid function tests during normal pregnancy [3,4]. We previously described the clinical variables that may modulate thyroid function within normal reference ranges, such as body mass index (BMI), smoking habits, or iodized salt consumption [5]. The healthy maternal thyroid adapts to this changing situation through corresponding changes in hormonal metabolism, iodine absorption, and regulation of the hypothalamic-pituitary–thyroid axis. For this reason, thyroid function tests in healthy pregnant women differ from those in non-pregnant women. 

The assessment of thyroid function in pregnant and non-pregnant people relies on the measurement of thyrotropin (TSH) and thyroid hormones [4]. Free thyroxine (FT4) shows a stronger correlation with thyroid status than total thyroxine (TT4). However, measurement of FT4 is a challenge, as a series of sources may bias the results, which applies even more during the gestational period. 

The increased specificity and the negligible influence of interferences in spectrometric methods make them superior to methods based on antigen–antibody recognition, such as immunoassays. However, as FT4 constitutes <0.02% of TT4, the use of spectrometric methods to detect low plasma FT4 concentrations was initially hampered insufficient sensitivity. Moreover, before applying the samples to liquid chromatography-mass spectrometry (LC/MS/MS) for quantitative analysis, they must first undergo an analytical step to physically separate the free from the protein-bound fraction (to albumin or TBG), which may be achieved by equilibrium dialysis (ED) or ultrafiltration (UF). In addition, recent guidelines (ATA 2017) recommend establishing local reference intervals for thyroid function tests to provide specific relevant normative data of the population in which they are to be used [4]. 

Thus, in this study, we aimed to establish trimester-specific reference ranges for TSH and FT4 in a cohort of healthy pregnant women in a population living in Catalonia, Spain. Since the FT4 reference interval (RI) in pregnancy varies widely between methods, we measured FT4 concentrations using two common automated commercial immunoassays in the three trimesters and using the reference LC/MSMS method in the first trimester (1T)**.**

## 2. Material and Methods

### 2.1. Study Design and Patients

We performed a prospective observational study including 339 healthy Caucasian pregnant women attending a primary pregnancy care center (ASSIR La Riera, Badalona, Spain), recruited during the 1T of pregnancy (before week 10 of gestational age) and followed up monthly to delivery. All women underwent an obstetric examination and fetal ultrasonography to confirm the normal progression of the pregnancy. In all cases, multivitamin supplementation, including iodine at a dose of 200 μg /day, was administered either before pregnancy or initiated at the first visit before week 10. Exclusion criteria were the presence of maternal and/or fetal disorders that might represent an obstetric or perinatal risk and women with a known history of family thyroid dysfunction; personal thyroid dysfunction; those who were taking thyroid hormone or antithyroid drugs; or those recently exposed to iodinated antiseptics. Clinical and nutritional data were obtained with a specific questionnaire that included the intake of iodized salt and dietary issues with iodine intake. In addition, all women provided blood samples during the three trimesters of gestation. All samples were stored at −80 °C until analyzed.

The Ethics Committee of the University Hospital Germans Trias i Pujol (HUGTiP) approved the study, and written consent was obtained from all the participants.

### 2.2. Laboratory Procedures

Biochemical parameters were measured using an AU58222 analyzer (Beckman Coulter, Fullerton, CA, USA). The reference ranges (RRs) were: 35–52 g/L for albumin, 48.6–90.2 µmol/L for creatinine, 5–35 U/L for alanine transaminase, and 30–400 µg/L for ferritin. Hematological parameters were measured using the Coulter VCS hematology analyzer (Beckman Coulter, Fullerton, CA, USA): the RR for women’s hemoglobin (Hb) was 7.95–9.93 mmol/L and 37–47% for hematocrit.

Architect^®^ Ref.7K78 (Abbott Diagnostic Division, Longford, Ireland) was used to measure total β-hCG; its LOD was <1.02 IU/L and CVs ranged between 1.6% and 4.9% for a concentration range between 24 and 5060 IU/L. The expected total hCG values for pregnant women at different gestational ages are (P 2.5-P 97.5): 1–10 weeks: 202–231,000 IU/L; 11–15 weeks: 22,536–234,990; 16–22 weeks: 8007–50,064; and 23–40 weeks: 1600–49,413 IU/L. 

Serum thyroglobulin was measured by a chemiluminescent electromagnetic immunoassay (ICMA) with a Cobas^®^ analyzer (Ref.06445896190, Elecsys^®^ Roche Diagnostics GmbH, Sandhofer Strasse 116, D-68305 Mannheim). Its LoQ was 0.1 µg/L and total CVs were <5.9%. The RR for the general population is 3.5–77 µg/L.

The Abbott Architect automated chemiluminescence immunoassay (CLIA) analyzer was used to measure serum TSH (Architect TSH^®^ Ref. 7K62, Abbott Diagnostic Division, Longford, Ireland). The limit of quantitation for TSH (LoQ) was <0.0038 mIU/L. Total precision resulted in CVs between 1.7% and 5.3% for a concentration range between 0.09 and 16.3 mIU/L. The manufacturer’s recommended TSH reference range for the general population is 0.35–4.54 mIU/L. For anti-thyroid peroxidase antibodies (TPOAb) (Architect Ref.2K47, Abbott Diagnostic Division, Longford, Ireland), the sensitivity of the assay was 0.16 IU/mL, LoQ was <0.50 IU/mL, and total CVs were <5%. A TPOAb concentration <5.61 IU/mL, corresponding to the 97.5th percentile of the reference interval for the euthyroid population, was regarded as TPO-negative. For anti-thyroglobulin antibodies (TgAb) (Architect Ref. 2K46, Abbott Diagnostic Division, Longford, Ireland), the sensitivity of the assay was 0.16 IU/mL, LoQ was <1.0 IU/mL, and total CVs were <5.9%. The reference range for the general population is <4.11 IU/mL.

FT4 was measured in the three trimesters using the Architect^®^ CLIA (FT4 Ref.7K65, Abbott Diagnostic Division, Longford, Ireland) and Cobas^®^ CLIA analyzers (Ref. 06,437,281 190, Elecsys^®^ Roche Diagnostics GmbH, Sandhofer Strasse 116, D-68305 Mannheim). For Architect^®^ CLIA, LoQ < 5.15 pmol/L, the total precision resulted in CVs between 3.6% and 7.8% for a concentration range between 8.9 and 38.7 pmol/L, and the RR for the general population was 9.0–19.0 pmol/L. For Cobas^®^ CLIA, the LoQ was 2.96 pmol/L, the total CVs was <6.3%, and the RR for the general population was 12–21.9 pmol/L. The RRs provided from the manufacturer during pregnancy are 12.1–19.6 pmol/L during 1T, second trimester (2T): 9.97–17.0 pmol/L, and third trimester (3T): 8.38–15.6 pmol/L (Document Reference Ranges Roche No.: 04640292). FT4 was also measured by LC/MS/MS during the 1T, according to Soldin et al. [6,7]. 

### 2.3. Liquid Chromatography-Mass Spectrometry (LC/MS/MS)

#### 2.3.1. Chemicals, Reagents, and Standards

Chromatography solvents water, acetonitrile, methanol, ammonium hydroxide, formic acid, and Centrifree YM-30 filters were supplied by Merck (Darmstadt, Germany). Thyroxine was obtained from Sigma-Aldrich (St Louis, MO; USA) (IRMM 468 European Commission Certified). The internal standard (IS) was 13C6-thyroxine obtained from Toronto Research Chemicals T425602 (Toronto, ON, Canada).

#### 2.3.2. Solutions and Standards

Stock solution of T4 was prepared using 40% ammonium hydroxide (*v*/*v*) in methanol to 12.872 mmol/L stored at −80 °C. The standards for T4 calibration points were 0.0, 1.28, 3.22, 6.44, 12.87. and 25.74 pmol/L, and the IS in methanol was 1.29 nmol/L.

#### 2.3.3. Sample Preparation

Five hundred microliters of serum was accurately deposited in Centrifree YM-30 (Merck, Darmstadt, Germany) filter tubes and warmed in a rack heated at 26 °C for 30 min. A Fiberlite 12 × 50 rotor was tempered in a Sorval RC 6 plus centrifuge (Thermo Scientific, Waltham, MA, USA) and held at 26 °C. The filters were then loaded with the samples and centrifuged at 3716 rpm at 26 °C for 2 h. More than 300 μL protein-free ultra-filtrate was obtained, and 300 μL was transferred in another tube, we added 12 μL of IS at 1.29 nmol/L, and the tube was vortexed for 1 min and let stand for one hour to balance with the matrix at 4 °C. Fifty microliters was injected into the C-18 column of the LC/MS/MS system.

#### 2.3.4. LC/MS/MS Setup

LC/MS/MS analysis was performed using an Agilent UHPLC 1290 Infinity II Series coupled to an Agilent QQQ/MS 6490 Series (Agilent Technologies, Santa Clara, CA, USA). Chromatographic separation was performed using an ACQUITY UPLC BEH C18 column (1.7 μm; 2.1 × 100 mm; Waters, Milford, MA, USA). The acetic acid in water was produced as acetonitrile (95:5, *v*/*v*) (solvent A) and acetonitrile:water (95:5, *v*/*v*) (solvent B). The flow rate was 0.3 mL/min, the injection volume was 50 µL, and the column temperature was set to 25 °C. The triple quadrupole was operated in ESI+ mode. The transitions used for each compound were *m/z* 777.7 > 731.7 and 777.7 > 604.9 for thyroxine and 783.7 > 737.7 for thyroxine-13C6.

The method validation parameters for free T4 (reproducibility, repeatability, accuracy, linear range, LOQ, and LOD) were evaluated. Overall, intraday (n = 5) and interday (n = 3) precisions were less than 7.2% and 12.6%, respectively, and accuracy was between 82.8% and 110.5%. The linear range was between the LOQ (3.86 pmol/L) and 25.74 pmol/L, and LOD was 1.29 pmol/L.

### 2.4. Statistics Analysis

Data were first tested for normal distribution using the Kolmogorov–Smirnov test to apply the appropriate analysis. Quantitative data are expressed as the mean (SD) and/or median (2.5th and 97.5th percentiles) as appropriate. The non-parametrical Friedman test was used to compare the continuous variables among the three trimesters. Comparisons between the two groups were performed using the Wilcoxon rank test. The chi-square test was applied to compare categorical variables. Correlations between variables were tested using the univariate Spearman’s correlation test. Comparisons of FT4 measurement methods were performed using the Passing-Bablok regression test [8]. Reference intervals (RIs) for thyroid function tests (95%, double-sided) and their corresponding 90% confidence intervals (CIs) were determined by the non-parametric percentile method according to the NCCLS and Clinical and Laboratory Standards Institute (CLSI) guidelines C28-A3 [9]. Statistical analyses were conducted with the statistical software package SPSS, version 17.0 (SPSS, Chicago, IL, USA) and MedCalc Statistical Software version 19.1.7 (MedCalc Software Ltd., Ostend, Belgium; https://www.medcalc.org/; accessed on 1 December 2020).

## 3. Results

A total of 32 women (10.6%) in the 1T, 19 (8.3%) in the 2T, and 17 (7.5%) in the 3T had positive TPO-Ab (titer > 5.61 IU/mL) and were excluded from the study for the calculation of RI, as evidence supports that it adversely modulates the impact of maternal thyroid status on the pregnancy and the developing fetus. Table 1 shows the clinical and biochemical data obtained in the final negative TPO-Ab cohort for the three pregnancy trimesters.

Statistically, all measured parameters showed differences throughout pregnancy. Urinary iodine progressively increased from the 1T to the 3T. In contrast, albumin, creatinine, ALT, ferritin, thyroglobulin, and hemoglobin concentrations and the percentage hematocrit showed a progressive decrease throughout pregnancy. No differences were found in TPO-Ab or Tg-Ab concentrations among the three trimesters.

Table 2 shows the trimester-specific RIs obtained in our TPO-Ab-negative population. The lower TSH limit showed a progressive increase from the 1T to the 3T. TSH limits in the 1T was significantly lower than in the 2T (*p* < 0.0001), and TSH limits in the 3T was higher than in the 2T (*p* < 0.0067).. FT4 limits showed a progressive decrease throughout pregnancy irrespective of the immunoassay method used. FT4 limits significantly decreased from 1T to 2T (either with Architect^®^: *p* < 0.0001 or with Cobas^®^: *p* < 0.0001) and from the 2T to the 3T (either with Architect^®^: *p* < 0.0001 or with Cobas^®^: *p* = 0.0067). The lower FT4 limit measured by Cobas^®^ was significantly higher than that measured by FT4 Architect^®^ and FT4 ID-LC/MS/MS in the 1T.

**Figure 1:**
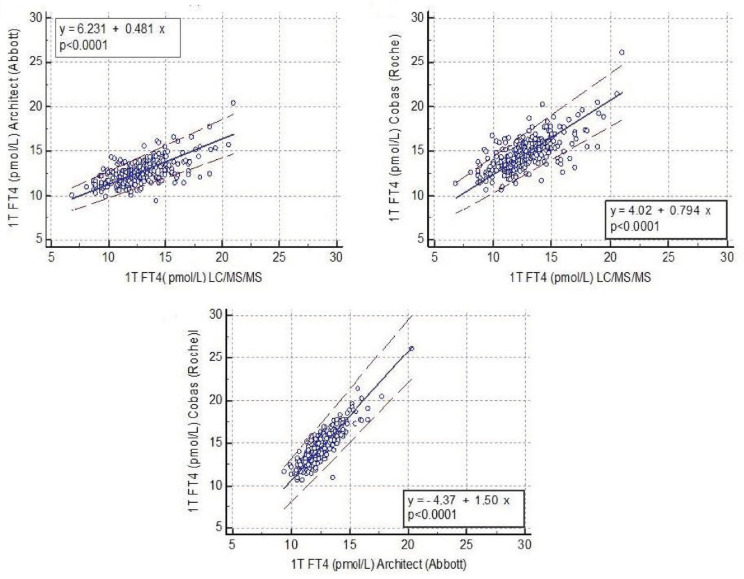
Correlations between free thyroxine concentrations measured by two immunoassays and liquid chromatography/tandem mass spectrometry.

Despite the FT4 results measured by LC/MS/MS with the two immunoassays being significantly correlated (*p* < 0.001 vs. FT4 Architect^®^; *p* < 0.001 vs. FT4 Cobas^®^), when applying the Passing-Bablock regression analysis, which is the statistical method for non-parametric regression analysis suitable for method-comparison studies, the results obtained by the three different methods were found to be non-interchangeable. The 95% confidence interval (CI) of the slopes in the regression in all comparisons did not include the digit 1 (slope Architect^®^ vs. LC/MS/MS: 0.481 (95% CI: 0.417–0.548); slope Cobas^®^ vs. LC/MS/MS: 0.794 (95%CI: 0.706–0.889); slope Architect^®^ vs. Cobas^®^: 1.5 (95% CI: 1.4–1.625) (Figure 1).

Table 3 shows the RI of FT4 measured by ED isotope dilution (ID)-LC/MS/MS published in the literature. The FT4 results measured by ED ID-LC/MS/MS by Anckaert et al. [10] are slightly higher than FT4 results reported by Kahric-Janici et al. and ours using UF ID-LC/MS/MS. However, our results show a remarkable concordance with those published by Soldin [11].

In the 1T, log-transformed TSH (LnTSH) showed a significant inverse correlation with the log-transformed FT4 (LnFT4) measured by the three methods (LnFT4 LC-MS/MS: r = −0.181, *p* = 0.002; LnFT4 Architect^®^: r = −0.254, *p* < 0.001; LnFT4 Cobas^®^: r: −0.280, *p* < 0.001). However, in the 2T and the 3T, LnTSH concentrations did not correlate with LnFT4 (either with Architect^®^ or Cobas^®^). Additionally, FT4 in the 1T measured by Cobas^®^ (Roche) and Architect^®^ (Abbot) significantly correlated with total β-hCG (r = 0.203, *p* < 0.001 and r = 0.315, *p* < 0.001, respectively, whereas β-hCG showed a weak negative correlation with TSH (r = −0.192, *p* = 0.002).

## 4. Discussion

In this prospective study, we defined trimester-specific RIs for TSH and FT4 by employing two of the most frequently used immunoassays in a large population of healthy pregnant women in Catalonia, in the northeast of Spain. In addition, we established normative values for FT4 at the 1T of pregnancy using ID-LC/MS/MS after a UF separation step.

The sample size used in the present study is sufficiently large for these RI to represent the reference population. Furthermore, our study is longitudinal, which means that the same women were evaluated in each of the three trimesters, and not cross-sectional, which additionally reinforces the data to be conclusive.

In agreement with others, the TSH values measured using the immunoassays significantly increased throughout pregnancy [12,13], while FT4 progressively decreased. Analogue-based FT4 immunoassays, which are flawed due to the special physiological conditions inherent in pregnancy, have significantly improved in the last decade. In a rigorous study comparing FT4 results obtained by currently used immunoassays to those provided by ED ID-LC/MS/MS, Anckaert et al. concluded that the immunoassay produced values are suitable for clinical evaluation of thyroid function during pregnancy [10], provided that proper RIs are available for the three trimesters. However, they found that results obtained with Architect^®^ (Abbott) did not show the same pattern as that observed using ID-LC/MS/MS or with Cobas^®^ (Roche), as the decrease in the 2T and 3T was less pronounced by 15% and 24%, respectively. This observation was attributed to the higher sensitivity of Architect^®^ to altered binding proteins during pregnancy. However, their study included a limited number of samples in the different trimesters. In our study, with more than 200 women in each trimester, the results from Architect^®^ displayed a similar pattern to those from Cobas^®^. The reference intervals of FT4 concentrations obtained by either Cobas^®^ and Architect^®^ agreed remarkably with those published for each immunoassay in large studies compared with Roche [12,14,15,16,17] and Abbott immunoassays [18]. Due to the known influence of ethnic variations on the thyroid function parameters, we only considered comparisons with studies in the European Caucasian population [19].

Due to the importance of verifying the reliability of FT4 immunoassay results with those obtained by the reference method, we also measured FT4 in the 1T using ID-LC/MS/MS, as this time point is considered most relevant in terms of physiological consequences for fetal neurodevelopment. This methodology provides the optimal characteristics with which to measure thyroid hormones in plasma due to the specificity, precision, and reliability of this methodology. However, to determine FT4 concentrations, serum samples must undergo a prior step to physically separate the free fraction from the protein-bound fraction (to albumin or TBG) by ED or UF, before subjecting the samples to LC/MS/MS for quantitative analysis [20]. The International Federation of Clinical Chemistry and Laboratory Medicine (IFCC) has recommended equilibrium dialysis combined with the determination of FT4 in the dialysate with LC/MS/MS [21,22] as the reference procedure. Soldin et al. [6] were the first to report the use of a UF procedure as the separation step prior to LC/MS/MS. UF is more suitable for clinical laboratories and replaces the expensive and time-consuming equilibrium dialysis with a rapid and reliable method that has been validated by this group and others versus ED LC/MS/MS [23]. In our study, we chose the ultracentrifugation method to separate free from protein-bound T4. We also applied the FC stipulated by the filter manufacturer and centrifugation temperature at 26 °C, according to Soldin [6].

Few studies have reported the RI of FT4 by LC/MS/MS in pregnant women. However, none have included more than 200 healthy women in the 1T of pregnancy, and our obtained results are similar to those previously published [10,11]. Moreover, the slope of the regression between FT4 Architect^®^ and UF ID-LC/MS/MS in our study was 0.481, close to the slope of 0.516 reported by Ankaert et al. [10] between these two methods. Similarly, the slope between FT4 measured by Cobas^®^ vs. UF ID-LC/MS/MS in our study was 0.779, matching the slope of 0.794 reported by them in their validation using the IFCC as the reference method. Yue et al. [24] also reported FT4 reference values measured by ID-LC/MS/MS in a large cohort of women in the 2T of pregnancy. To the best of our knowledge, no further studies have reported an FT4 RI measured by LC/MS/MS during pregnancy.

In our study, median FT4 concentrations measured by LC/MS/MS in the 1T were not significantly different than FT4 measured using the Architect^®^ method. In contrast, the median FT4 measured by Cobas^®^ was significantly higher than those of LC/MS/MS and from Architect^®^. However, the Passing-Bablok regression analysis, the statistical method used for non-parametric regression analysis that is suitable for method-comparison studies [8], proved that the RIs obtained by each of the three methods were not interchangeable. Thus, the RI must be determined for every one of the different immunoassays. However, as previously stated, FT4 concentrations measured with analogue immunoassays may not be as reliable for samples with FT4 concentrations above and below the reference range. Thus, it is crucial, as in our study, to have a reference method available to verify results in patients in which thyroid dysfunction may be suspected [23,24,25,26,27,28].

A limitation of the study is that we could not recruit a multiethnic cohort or participants who were not consuming iodine supplements, so our reference ranges are based on Caucasian pregnant women with an optimal iodine status (although based on iodine supplements). Another limitation of the study is that we performed FT4 analyses by LC/MS/MS only in the 1T of pregnancy. Although the 1T is the one that has the most clinical implications for the fetus, it would have been interesting to perform FT4 analyses by LC/MS/MS in the 2T and the 3T, and compare the results with those performed by the two immunoassays methods.

## 5. Conclusions

We established trimester-specific RIs for our population with two automated analogue immunoassays, which seem adequate for clinical follow-up for most pregnant women. In addition, we also validated the measurement of FT4 using ID-LC/MS/MS after UF physical separation to confirm the results of FT4 lower than the 2.5th percentile or higher than the 97.5th percentile using a gold-standard method to ensure the presence of thyroid dysfunction in these patients.

## Figures and Tables

**Table 1:** Clinical and biochemical parameters in the negative TPO cohort at the three trimesters of pregnancy.

	First Trimester	Second Trimester	Third Trimester	*p* ^#^
•Number	270	212	211	
•Maternal age (years) *^&^	32.3 ± 5.2			
•Maternal weight (kg) *^&^	64.5 ± 13.6			
•Maternal BMI (kg/m^2^) *^&^	24.8 ± 4.9			
•Parity (%)				
-First gestation	48			
-Second gestation	41			
-Third or more gestation	11			
•Previous miscarriages (%)				
-None	61			
-One	29			
-Two or more	10			
•Gestational age (weeks) ^&&^	9–11	24–28	29–33	
•Level of education (%)				
-None/primary	25.3			
-Secondary	49.6			
-Higher education	25.1			
•Smoking habit (%) ^&^				
-Nonsmoker	80.2			
-Smoker	18.2			
•Working women (%)	81.2			
•Consumption of iodized salt (%) ^&^	25.3%			
•Use of supplements (%) ^&^				
-None	46			
-Potassium iodide	28.6			
-Multivitamins	25.4			
TPO-Ab ** (UI/mL)	0.55 (0.5–1.37)	0.57 (0.5–3.09)	0.55 (0.5–2.66)	NS
Tg-Ab (UI/mL) **	0.99 (0.4–9.7)	1.04 (0.43–7.0)	0.99 (0.38–5.4)	N.S:
HCG (mUI/mL) **	110,583 (20,164–345,434			
Albumin (g/L) **	40.2 (30.5–45)	33.9 (30.3–38.4) ^a^	32.9 (29.1–37) ^a,b^	<0.001
Creatinine ** (mg/dL)	0.56 (0.43–0.73)	0.52 (0.38–0.70) ^a^	0.5 (0.4–0.7^a,b^	<0.001
ALT (U/L) **	12 (6.58–40.85)	12.0 (6.0–37.8)	11 (6.0–50)	0.129
Ferritin (ng/mL) **	42 (9–170)	11.0 (4.8–80.5) ^a^	13 (4–46.8)^a, b^	<0.001
Thyroglobulin (ng/mL) **	15.3 (1.9–72.2)	12.3 (1.9–81.1) ^a^	13.5 (2.4–86.6) ^a,b^	<0.001
Urinary Iodide (µg/L) **	126.2 (32–402.3)	178 (51.6–547) ^a^	170 (37.5–543) ^a,b^	<0.001
Hb (g/dL) **	12.8 (10.9–14.5)	11.4 (9.3–13.2) ^a^	11.6 (10.1–13.6) ^a,b^	<0.001
Ht (%) **	37.9 (32.4–42.8)	33.4 (27.9–38.6) ^a^	34.5 (29.9–40.5) ^a,b^	<0.001

BMI: body mass index; TPO-Ab: anti-thyroid peroxidase antibodies; Tg-Ab: anti-thyroglobulin antibodies; HCG: total human chorionic gonadotropin; Hb: hemoglobin; Ht: hematocrit. * Mean ± standard deviation; ** Median and 95% range (2.5th–97.5th percentiles). ^&^ at time of recruitment; ^&&^ adjusted by fetal ultrasonography at the first visit. ^#^ significance among 3 groups of related patients (1T, 2T, and 3T) using the non-parametrical Friedman test. Pairwise differences according to the Wilcoxon rank test: ^a^ *p* < 0.0001 vs. 1st Trimester; ^b^ *p* < 0.0001 vs. 2nd Trimester.

**Table 2:** The 95% reference interval, double-sided, determined by the non-parametric percentile method (CLSI C28-A3) for thyrotropin and free thyroxine in the 3 trimesters of pregnancy, measured by immunoassays, and free thyroxine, measured by liquid chromatography/tandem mass spectrometry (in the first trimester).

	First Trimester	Second Trimester	Third Trimester
	(*n* = 270)	(*n* = 212)	(*n* = 211)
TSH Architect^®^			
Lower limit (90% CI)	0.03 (0.02 to 0.11) ^a^	0.51 (0.34 to 0.62) ^b^	0.50 (0.38 to 0.67)
Upper limit (90% CI)	3.78 (3.26 to 4.71)	3.53 (3.26 to 4.06)	4.32 (3.52 to 4.72)
FT4 Architect^®^			
Lower limit (90% CI)	10.42 (10.04 to 10.55) ^a^	8.37 (8.37 to 8.88) ^b^ 12.74 (12.3.6 to 13.13)	8.24 (7.85 to 8.62)
Upper limit (90% CI)	15.96 (15.32 to 16.6)		12.49 (12.23 to 13.0)
FT4 Cobas^®^			
Lower limit (90% CI)	11.46 (10.94 to 11.58) ^a,^*	9.65 (8.88 to 9.91) ^c^	8.88 (8.11 to 9.27)
Upper limit (90% CI)	19.05 (18.28 to 20.47)	14.67 (14.16 to 15.83)	14.54 (14.16 to 15.32)
FT4 ID-LC/MS/MS		-	-
Lower limit (90% CI)	8.75 (8.75 to 9.27)		
Upper limit (90% CI)	18.27(17.12 to 19.44)		

TSH: thyrotropin (µUI/mL); FT4: free thyroxine (pmol/L); ID-LC/MS/MS: isotope dilution-liquid chromatography/tandem mass spectrometry. CI: confidence interval. ^a^ *p* < 0.0001 compared with the second trimester; ^b^ *p* < 0.0001 compared with the third trimester; ^c^ *p* < 0.0067 compared with the third trimester; * *p* < 0.0001 compared with FT4 Architect^®^ and with FT4 ID-LC/MS/MS.

**Table 3:** Reference intervals of free thyroxine measured by liquid chromatography/tandem mass spectrometry published by different studies.

	First Trimester			Second Trimester			Third Trimester		
Authors	n	w	FT4 (pmol/L)	n	w	FT4 (pmol/L)	n	w	FT4 (pmol/L)
Hernandez JM et al. 2020	270	<10	8.75–18.27 *						
Anckaert E et al. 2010 (10)	29	12.6 ± 0.6	16.8 ± 2.2 ^#^ 12.49–21.1 **	33	25.3 ± 2.2	13.0 ± 1.8 ^#^ 12–14.2 **	34	36.1 ± 1.1	13.0 ± 2.3 ^#^ 11.0–14.8 **
Kahric-Janinc et al. 2007 (11)	59	8.7	14.6 ± 2.96^#^ 8.8–20.4 **	35	17.8	11.9 ± 3.9 ^#^ 4.3–19.5 **	26	28.7	11.1 ± 2.71 ^#^ 5.8–16.4 **
Yue et al. 2008 (24)				72 120	14 20	13.9–15.2 * 11.1–19.7 *			

w: weeks of pregnancy; FT4: free thyroxine. * Reference limits of 95% confidence intervals measured by the non-parametric method (CLSI C28-A3); ** 95% range (mean ± 1.96 standard deviation). ^#^ mean ± standard deviation.
